# Correlation of Body Mass Index with Oncologic Outcomes in Colorectal Cancer Patients: A Large Population-Based Study

**DOI:** 10.3390/cancers13143592

**Published:** 2021-07-17

**Authors:** Chong-Chi Chiu, Chung-Han Ho, Chao-Ming Hung, Chien-Ming Chao, Chih-Cheng Lai, Chin-Ming Chen, Kuang-Ming Liao, Jhi-Joung Wang, Yu-Cih Wu, Hon-Yi Shi, Po-Huang Lee, Hui-Ming Lee, Li-Ren Yeh, Tien-Chou Soong, Shyh-Ren Chiang, Kuo-Chen Cheng

**Affiliations:** 1 Department of General Surgery, E-Da Cancer Hospital, Kaohsiung 82445, Taiwan; chiuchongchi@gmail.com (C.-C.C.); ed100647@edah.org.tw (C.-M.H.); huiming00@gmail.com (H.-M.L.); 2 School of Medicine, College of Medicine, I-Shou University, Kaohsiung 82445, Taiwan; 3 Department of Medical Research, Chi Mei Medical Center, Tainan 71004, Taiwan; ho.c.hank@gmail.com (C.-H.H.); cih830927@gmail.com (Y.-C.W.); 4 Cancer Center, Wan Fang Hospital, Taipei Medical University, Taipei 11695, Taiwan; 5 Department of Information Management, Southern Taiwan University of Science and Technology, Tainan 71005, Taiwan; 6 College of Medicine, I-Shou University, Kaohsiung 82445, Taiwan; pohuang1115@ntu.edu.tw (P.-H.L.); tienchou0117@gmail.com (T.-C.S.); 7 Department of Intensive Care Medicine, Chi Mei Medical Center, Liouying 73657, Taiwan; ccm870958@yahoo.com.tw; 8 Department of Dental Laboratory Technology, Min-Hwei College of Health Care Management, Tainan 73657, Taiwan; 9 Department of Internal Medicine, Kaohsiung Veterans General Hospital, Tainan Branch, Tainan 71004, Taiwan; dtmed141@gmail.com; 10 Department of Intensive Care Medicine, Chi-Mei Medical Center, Tainan 71004, Taiwan; chencm3383@yahoo.com.tw; 11 Department of Internal Medicine, Chi Mei Medical Center, Chiali 72263, Taiwan; abc8870@yahoo.com.tw; 12 Department of Anesthesiology, Chi Mei Medical Center, Tainan 71004, Taiwan; 400002@mail.chimei.org.tw; 13 Department of Anesthesiology, National Defense Medical Center, Taipei 11490, Taiwan; 14 Department of Healthcare Administration and Medical Informatics, Kaohsiung Medical University, Kaohsiung 80708, Taiwan; hshi@kmu.edu.tw; 15 Department of Business Management, National Sun Yat-Sen University, Kaohsiung 80424, Taiwan; 16 Department of Medical Research, Kaohsiung Medical University Hospital, Kaohsiung 80756, Taiwan; 17 Department of Medical Research, China Medical University Hospital, China Medical University, Taichung 40402, Taiwan; 18 Department of Surgery, E-Da Hospital, Kaohsiung 82445, Taiwan; 19 Department of Anesthesiology, E-Da Cancer Hospital, Kaohsiung 82445, Taiwan; ed110880@edah.org.tw; 20 Department of Medical Imaging and Radiology, Shu-Zen Junior College of Medicine and Management, Kaohsiung 82144, Taiwan; 21 Weight Loss and Health Management Center, E-Da Dachang Hospital, Kaohsiung 80794, Taiwan; 22 Department of Internal Medicine, Chi Mei Medical Center, Tainan 71004, Taiwan; crsmed@yahoo.com.tw; 23 Department of General Education, Chia Nan University of Pharmacy and Science, Tainan 71710, Taiwan; 24 Department of Safety, Health and Environment, Chung Hwa University of Medical Technology, Tainan 71703, Taiwan

**Keywords:** colorectal cancer, body mass index, oncologic prognosis, overall survival, disease-free survival, colorectal cancer-specific survival

## Abstract

**Simple Summary:**

Obesity is related to the rising risk of colorectal cancer (CRC). However, the impact of body mass index (BMI) on the oncologic prognosis of CRC patients remains unknown. Conflicting results regarding the relationship between BMI and CRC prognosis have been reported. Therefore, we conducted a nationwide retrospective study that examined the correlation of BMI at diagnosis with overall survival (OS), disease-free survival (DFS), and CRC-specific survival rates in CRC patients. We noted that an underweight status at diagnosis was related to higher mortality and recurrence rates, a decreased rate of OS, and a decreased CRC-specific survival rate compared with those for the normal weight patients. In contrast, overweight and class I or II obese patients had better OS, CRC-specific survival, and DFS rates than those in the normal weight category. Our findings suggest that weight loss in the immediate diagnosis period is unwarranted.

**Abstract:**

It has been acknowledged that excess body weight increases the risk of colorectal cancer (CRC); however, there is little evidence on the impact of body mass index (BMI) on CRC patients’ long-term oncologic results in Asian populations. We studied the influence of BMI on overall survival (OS), disease-free survival (DFS), and CRC-specific survival rates in CRC patients from the administrative claims datasets of Taiwan using the Kaplan–Meier survival curves and the log-rank test to estimate the statistical differences among BMI groups. Underweight patients (<18.50 kg/m^2^) presented higher mortality (56.40%) and recurrence (5.34%) rates. Besides this, they had worse OS (aHR:1.61; 95% CI: 1.53–1.70; *p*-value: < 0.0001) and CRC-specific survival (aHR:1.52; 95% CI: 1.43–1.62; *p*-value: < 0.0001) rates compared with those of normal weight patients (18.50–24.99 kg/m^2^). On the contrary, CRC patients belonging to the overweight (25.00–29.99 kg/m^2^), class I obesity (30.00–34.99 kg/m^2^), and class II obesity (≥35.00 kg/m^2^) categories had better OS, DFS, and CRC-specific survival rates in the analysis than the patients in the normal weight category. Overweight patients consistently had the lowest mortality rate after a CRC diagnosis. The associations with being underweight may reflect a reverse causation. CRC patients should maintain a long-term healthy body weight.

## 1. Introduction

Colorectal cancer (CRC) is among the leading causes of cancer death worldwide, with an approximate incidence of 0.02% [1]. There are about 1.2 million newly diagnosed CRC patients every year [2]. The increasing incidence of CRC in East Asian countries has been attributed to changes in diet and lifestyle and the popularization of cancer screening in recent decades [3].

Obesity involves excessive body fat accumulation and, therefore, it increases the risk of metabolic syndrome [4]. It is a significant health concern worldwide. This global pandemic of obesity increases the risks of multiple cancer occurrence, including CRC [5]. Besides this, the 5-year survival rate of CRC is still disappointing, especially in developing areas [6]. Indeed, it is reasonable to pay attention to CRC prevention.

The body mass index (BMI) is the most popular scale to assess obesity [7]. A meta-analysis study including 41 reports found that obesity increased CRC risk by 33% among 8,115,689 people [8]. Baade et al. [9] and Kuiper et al. [10] demonstrated that overweight CRC patients had improved CRC-related mortality rates by 25% and 55%, respectively. However, Meyerhardt et al. showed no disparity in the mortality risk between overweight and normal weight CRC patients [11,12]. These conflicting results have also been found among studies that examined the correlation between obesity and CRC prognosis. Conversely, some studies have revealed that being underweight is related to an increased risk of mortality. This phenomenon is probably caused by cancer progression-associated weight loss [9,11,12,13]. Realizing the relationship between BMI and CRC prognosis is highly important to provide body weight guidelines for CRC patients [14].

Most studies about the relationship between obesity and a CRC oncologic prognosis were performed in European institutions and have seldom been performed in East Asian areas. Besides this, East Asian people generally have a lower ratio of overall obesity but are more centrally obese than Europeans. Some studies have demonstrated that Asian people have a comparatively higher visceral fat ratio than Europeans with the same BMI. Thus, Asian people are more vulnerable to insulin resistance [15]. From the literature, only four studies have investigated the impact of obesity on CRC oncologic prognoses among Asian people [16,17,18,19]. However, some demerits of these studies include the small sample sizes, the lack of investigation into the correlation between central obesity and CRC survival rates, and the lack of potential confounder adjustment [20]. This study was performed to investigate the clinical significance of BMI and oncologic outcomes using a large nationwide CRC patient cohort from Taiwan.

## 2. Materials and Methods

### 2.1. Study Design and Data Sources

The administrative claims datasets, including the national health insurance research database (NHIRD), the Taiwan Cancer Registry (TCR), and Taiwan’s cause of death dataset, from the Health and Welfare Data Science Center (HWDC), Ministry of Health and Welfare (MOHW) were used in this study. The HWDC is a data center that integrates all health-related datasets for research purposes. The NHIRD is based on Taiwan’s National Health Insurance program, which covers more than 23 million people. The NHIRD is comprised of detailed individual information, including age, sex, diagnosis date, disease and procedure codes, prescription, and reimbursement claims. Besides this, the TCR was built in 1979 to monitor Taiwan’s cancer incidence, and the mortality rates were used to identify cancer patients. The basic information within the TCR includes patient demographics, clinical stages of cancer, cancer primary sites, tumor histology, and treatment types. To add more precise diagnosis and treatment items, in 2002 the TCR established long-form datasets for oncology categories such as colon and rectum [21].

The data for this study were retrieved from the TCR. The claims of diagnosis and the inpatient and outpatient information of patients were obtained from the NHIRD. Diagnosis codes were selected from the International Classification of Diseases, Ninth Revision, Clinical Modification (ICD-9-CM). This study was exempt from the institution’s internal review board’s full review because our secondary data analysis could not identify the patients. However, the study protocol conformed to the 1964 Declaration of Helsinki’s ethical standards.

### 2.2. Study Patients

**Figure 1:**
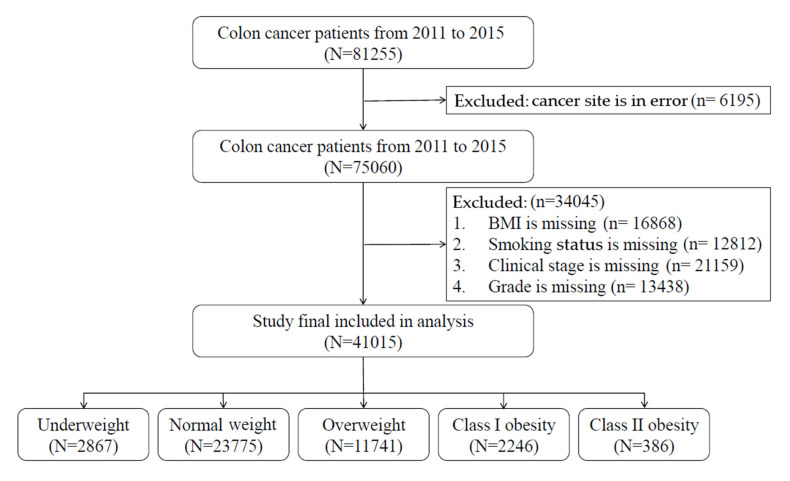
Flowchart of the recruitment and study procedure.

The TCR included all patients with information retrieved from the International Classification of Diseases for Oncology, third edition (ICD-O-3) with colon (ICD-O-3: C18), rectosigmoid junction (ICD-O-3: C19), and rectum (ICD-O-3: C20) as the primary cancer from January 2011 to December 2015. To exclude potential misclassification, the cases without a correct diagnosis date, information on the clinical stage, treatment type, body weight, or body height at the time of cancer diagnosis were excluded. A diagram illustrating the selection of study patients is provided in Figure 1.

### 2.3. Measurements

The study variables, including age at diagnosis, gender, clinical stage, histology grade, treatment type, and smoking status, were all collected from the TCR. The age group was divided into the categories <40, 40–49, 50–59, 60–69, and ≥70. Smoking status categories were defined as patients who have never smoked, have ever smoked (since quit), and currently smoke. As some comorbidities may result in premature mortality not related to the primary colon or rectum malignancies but may be closely associated with obesity, we also analyzed the effect of these comorbidities, including myocardial infarction (ICD-9-CM: 410, 412), congestive heart failure (ICD-9-CM: 428), peripheral vascular disease (ICD-9-CM: 441, 443.9, 785.4), dementia (ICD-9-CM: 290,294, 331), chronic obstructive pulmonary disease (ICD-9-CM: 490–496, 500–505, 506.4), rheumatic disease (ICD-9-CM: 725, 710.0–710.1, 710.4, 714.0–714.2, 714.81, 517.1), peptic ulcer disease (ICD-9-CM: 531–534), mild liver disease (ICD-9-CM: 571.2, 571.4–571.6), diabetes mellitus without chronic complications (ICD-9-CM: 250.0–250.3, 250.7), diabetes mellitus with chronic complications (ICD-9-CM: 250.4–250.6), hemiplegia or paraplegia (ICD-9-CM: 342, 344.1), renal disease (ICD-9-CM: 582–583, 585–586, 588), and moderate and severe liver diseases (ICD-9-CM: 456.0–456.2, 572.2–572.8).

We examined the following survival endpoints: overall survival (OS), defined as the time from pathologic diagnosis to death due to any reason, and disease-free survival (DFS), defined as the time from pathologic diagnosis to disease progression or first recurrence. Besides these, CRC-specific survival was also estimated as the cause of CRC death among those patients. BMI at diagnosis was defined as the BMI at the time of the initial cancer diagnosis. The BMI was categorized into the groups underweight (<18.50 kg/m^2^), normal weight (18.50–24.99 kg/m^2^), overweight (25.00–29.99 kg/m^2^), class I obesity (30.00–34.99 kg/m^2^), and class II obesity (≥35.00 kg/m^2^) according to the 2000 World Health Organization (WHO) guidelines [22].

### 2.4. Statistical Analysis

Summary statistics were presented as the median (range) for continuous variables, and frequency (percentage) for categorical variables. For different BMI groups, continuous variables were analyzed using the Kruskal–Wallis test and categorical variables were analyzed using the Chi-squared test. The trend distributions of the OS, DFS, and CRC-specific survival rates were plotted using the Kaplan–Meier approach with the log-rank test to estimate the statistical differences among the different BMI groups.

The multivariable Cox proportional regression model was used to evaluate hazard ratios (HRs) with 95% confidence intervals (CIs) of OS, DFS, and CRC-specific survival rates. The HRs of OS, DFS, and CRC-specific survival rates were all presented as age-/gender-adjusted HRs, age-/gender-/cancer-stage-adjusted HRs, and adjusted HRs with all selected confounding factors, including age, gender, cancer stage, grade, cancer site, treatment types, smoking status, and comorbidities. A stratified analysis for age group, gender, clinical stage, and cancer site was also conducted. SAS 9.4 (SAS Institute, Inc., Cary, NC, USA) was used for statistical analysis. *p*-values for two-sided analyses were considered statistically significant at *p* < 0.05.

## 3. Results

### 3.1. Patient Characteristics

Among our 41,015 CRC patients, there were 57.97% normal weight, 6.99% underweight, 28.63% overweight, 5.47% class I obesity, and 0.94% class II obesity patients based on the international classification of BMI. Most CRC patients were older than 60 years old (age 60–69, 25.54%; age ≥ 70, 41.42%), and 56.64% were male. Demographic and baseline variables for all CRC patients and the different BMI groups are presented in Table 1. Patients who were underweight presented higher mortality (56.40%) and recurrence (5.34%) rates.

### 3.2. Hazard Ratios of BMI at Diagnosis and Mortality Rates

Table 2 shows that patients belonging to the underweight group had worse OS (aHR: 1.61; 95% CI: 1.53–1.70; *p*-value: <0.0001) and CRC-specific survival (aHR: 1.52; 95% CI: 1.43–1.62; *p*-value: <0.0001) rates than those patients of normal weight after adjusting for selected confounding factors. However, patients in the overweight, class I obesity, and class II obesity groups showed a statistically significant protective effect on OS and CRC-specific survival rates compared with those of normal weight. We also found a statistically significant risk of underweight BMI on DFS rates when compared to CRC patients with a normal weight.

### 3.3. Stratified Analyses of Overall Survival Rates

The stratified OS rates for age, gender, clinical stage, and cancer site are presented in Table 3. In patients aged <50 years, there were no significant differences between the normal weight group and the other four BMI groups. However, in those older than 50, a significantly lower OS rate was noted in the underweight group compared to the normal weight group. On the contrary, a significantly better OS rate was noted in the overweight group older than 50 and in the class I and II obesity groups older than 70 compared to that in the normal weight group. A worse OS rate was noted in underweight patients of both genders. A statistically significantly better OS rate was noted in patients from the overweight, class I obesity, and class II obesity groups, except in class II obese males. Considering the clinical stage factor, we also found the same adverse results for underweight patients of all stages. Nevertheless, we noted that the OS rate for stage 0 was lower than that for other stages, which might be related to the bias caused by a smaller patient number in that group. A worse OS rate was also noted in patients in the all-stage/overweight groups, stage 2/class I obese group, and stage 4/class II obese group.

### 3.4. Stratified Analyses of Disease-Free Survival Rates

Table 4 presents the stratified DFS rates. In patients aged <50 years, there was no significant difference between the normal weight group and the other four BMI category groups. However, a significantly worse DFS rate was noted in the underweight group than in the normal weight group, which was mainly present in the subgroup older than 50. On the contrary, a significantly better DFS rate was noted in the overweight group older than 50 and in the class I obesity group older than 70, a similar trend to the stratified OS rate in Table 3. A worse DFS rate was noted in underweight patients of both genders. A statistically significantly better DFS rate was noted in male patients in the overweight and class I obese groups. As for the clinical stage factor, adverse results were noted in the underweight patients in stages 0, 1, 2, and 3. Lower HRs were also noted in overweight patients in stages 0, 1, 2, and 3 and in class I obese patients in stages 0 and 2.

### 3.5. Stratified Analyses of Colorectal-Cancer-Specific Survival Rates

Table 5 presents the stratified CRC-specific survival estimates. In patients aged <50 years, there was no significant difference between the normal weight group and the other four BMI category groups. However, in those older than 50, the CRC-specific survival rate in the underweight group was significantly lower. Besides this, the CRC-specific survival rate was higher in overweight patients aged 50–59 or ≥70 years and in class I obese patients aged ≥70 years. For the gender factor, we noted a statistically significantly worse CRC-specific survival rate in the underweight patients and better CRC-specific survival rates in the overweight, class I obese, and class II obese patient groups. In relation to the clinical stage, adverse results were noted in all underweight patients. On the contrary, a protective effect was noted in patients in stages 1, 2, 3, and 4, class I obese patients in stage 2, and class II obese patients in stages 2 and 4.

## 4. Discussion

Globally, the adult population’s BMI has followed an increasing trend over the past 30 years [23]. According to the WHO’s statistical data, about 1.5 billion adults are overweight, and 500 million are obese [2]. Obesity is one of the proven risk factors for CRC development and death [24], but only recently has research investigated the effect of obesity on survival following a cancer diagnosis [25]. In other words, its association with oncologic outcomes among CRC patients is ambiguous, and conflicting results have been reported [26].

The mechanisms of the relationship between excess weight and CRC remain unclear. Several possibilities have been hypothesized [8] and primarily linked with obesity-related hormonal changes [14]. The insulin/insulin-like growth factor (IGF) axis and adipokines (adiponectin and leptin) are the two hormonal systems that have been investigated most thoroughly [8]. Obesity correlates with elevated insulin levels, free IGFs, and adipocyte-derived factors, including leptin, the tumor necrosis factor-alpha (TNF-α), and interleukin-6 (IL-6). Besides this, it is also related to adiponectin reduction [27]. These obesity-related hormonal changes have been noted to have a positive association with CRC incidence [28], but because of their interrelations, it has been hard to clarify their causal relationship [14]. However, an increased amount of circulating insulin and free IGF-I has been related to physical inactivity and obesity [29]. Coussens and Werb et al. remarked that obesity-induced chronic inflammation is associated with cancer occurrence [30]. Trevisan et al. also demonstrated that increasing obesity could cause insulin resistance [31], which was reported to have a triple risk of CRC-related mortality in their research involving 62,285 patients [32]. They confirmed that IGF-I and insulin could boost cell proliferation and impede apoptosis in CRC cells [33], suggesting that they may actuate the growth of micro-metastases [26].

However, another mechanism of various BMI levels leading to CRC survival differences is suggested by the evidence of fatty acid synthase (FASN) expression. In normal weight and minimally overweight patients (BMI < 27.5 kg/m^2^), FASN positivity was noted with a lower mortality rate, whereas among moderately overweight patients (BMI ≥ 27.5 kg/m^2^), overexpression of FASN increased the mortality rate significantly [34]. FASN essentially leads to de novo lipogenesis and is physiologically adjusted by energy balance. Besides this, both exercise and energy restriction downregulate FASN [35]. This phenomenon has raised the question of whether different severity levels of obesity would lead to conflicting oncologic outcomes during cancer management.

In the context of disease, overweight patients may have survival benefits attributed to a better nutritional status, more optimal medical treatment, more significant endothelial progenitor cells, lower thromboxane production, higher ghrelin sensitivity, and lower concentrations of TNF-α [36]. Overweight CRC patients might have more significant muscle and fat mass, empowering them to deal with tumor progression and the metabolic demands of treatment. However, even though mortality was lowest in the overweight group, the risk was similarly low in high-normal weight patients, suggesting that a BMI in this range may minimize the risks during cancer treatment [36]. In a multivariate analysis by Sinicrope et al. [26], better OS rates in overweight patients were shown. However, this phenomenon was only noted in men (*p* = 0.006) after adjusting for the factors of age, stage, and treatment strategies. Other specialists have also demonstrated that overweight patients with no cancer history have the lowest mortality risk [37,38]. However, this result may reveal a limitation of the BMI analysis because it cannot identify body weight from muscle versus fat; therefore, muscular patients could be categorized as overweight or obese, though they are not really. Of course, these findings that suggest that being overweight is related to a lower mortality rate might not necessarily be causal but rather they reveal that the reference (normal weight) group may include initially overweight patients who might have lost weight due to disease. This would mean increased patient numbers in the reference group with underlying diseases or weight lost due to CRC progression. As their BMI was recorded at the time of cancer diagnosis (BMI at diagnosis), a possible bias related to reverse causation has raised a concern.

Our series found that the higher BMI our patients had, the better OS rates they encountered (Table 2). However, Campbell et al. found a higher relative risk of all-cause mortality among patients with class II or III obesity (BMI of ≥35 or ≥40 kg/m^2^, respectively) [39]. It is essential to realize that one of the leading causes of mortality in these obese CRC patients was cardiovascular disease rather than cancer recurrence, leading to an increased overall mortality. However, Americans are more commonly obese (33.7%) than the Taiwanese (7.2%). Besides this, being very obese is much more common in the American population: 13.4% of the total U.S. sample and about 1% in Taiwan are obese. The average BMI values of the obese and very obese are higher in the American group than in the Taiwanese group [40], which might have led to this discrepancy.

In a prior study by Flegal et al. [41], mortality in underweight CRC patients was more often due to non-cancer-related causes than that in normal weight patients. This increased risk of mortality could also be associated with other underlying diseases such as advanced type two diabetes [42], cardiac failure [43], and pulmonary diseases [44]. As weight loss is one of the CRC symptoms, more advanced cancer patients would experience more significant weight loss and possibly be underweight at diagnosis [11]. Significant cancer-associated weight loss might identify a poorer prognostic subgroup because cancer cachexia leads to poorer outcomes [45]. The depletion of adipose tissue accounts for most cancer-related weight loss, yet the preferential loss of the skeletal muscle negatively impacts mortality [46].

**Figure 2:**
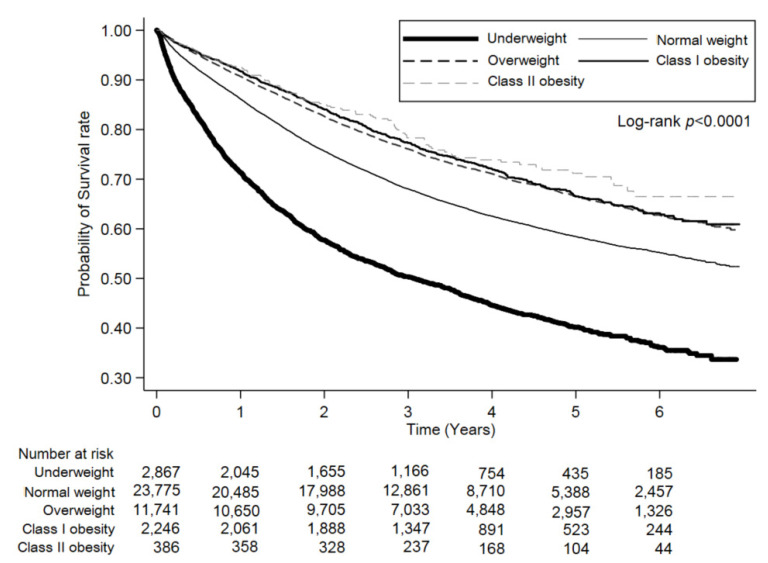
Kaplan–Meier curves for overall survival (OS) rates across the body mass index (BMI) categories.

**Figure 3:**
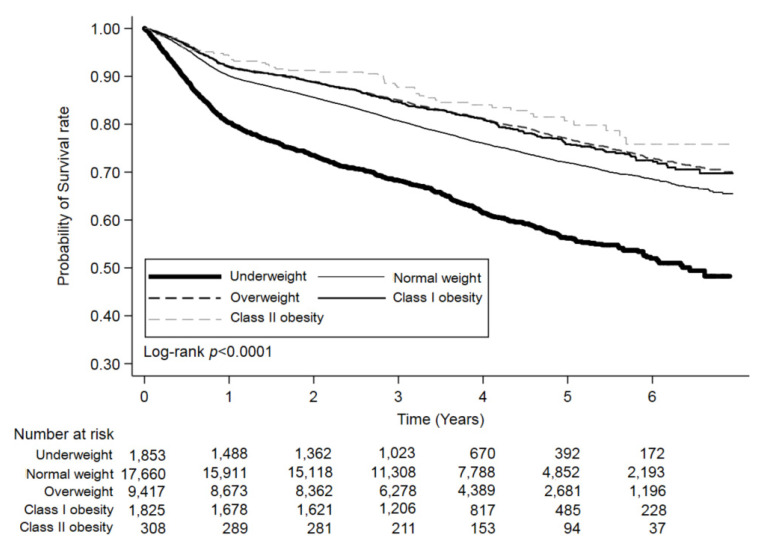
Kaplan–Meier curves for disease-free survival (DFS) rates across the body mass index (BMI) categories.

**Figure 4:**
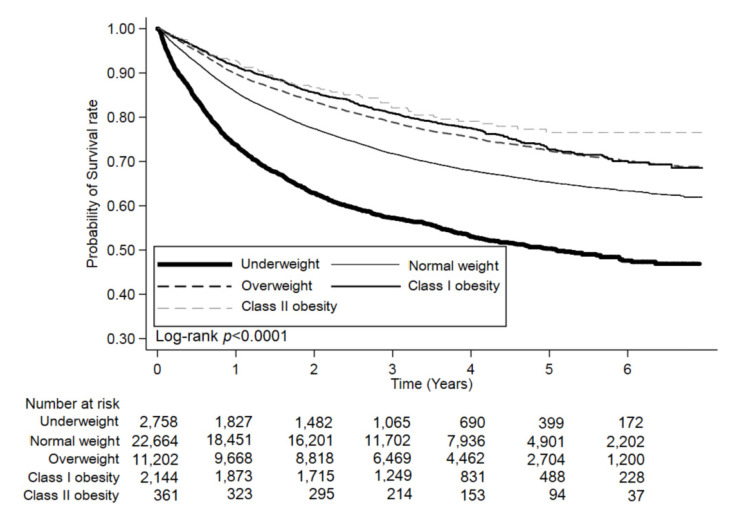
Kaplan–Meier curves for CRC-specific survival rates across the body mass index (BMI) categories.

Our study found that CRC-specific survival rates decreased among underweight patients (Table 2). This phenomenon was encountered after an adjusted analysis with three types of combinations (age with gender, age with gender and stage, and multivariate). These findings emphasize BMI as a decisive prognostic factor for CRC, with the underweight BMI category being disadvantageous for CRC patients. This result has been a consistent finding among the studies mentioned above. Another significant result was that underweight patients had increased cancer recurrence rates (Table 1) and poorer oncologic outcomes in a multivariate analysis (Table 2). The results of shorter OS, DFS, and CRC-specific survival rates for underweight patients suggested that the prognosis was also cancer-related (Table 3 and Figure 2, Table 4 and Figure 3, Table 5 and Figure 4). Our evidence shows that the time frame during which BMI is determined could affect its relationship to the clinical outcome. Since BMI was recorded at diagnosis and enrollment in our study, it is essential to differentiate underweight patients who had a stable weight over time from those who underwent significant cancer-related weight loss at diagnosis. However, some patients would intentionally lose weight through a healthier diet and exercise, whereas other patients experience weight loss because of cancer progression or treatment side effects. Our BMI at diagnosis data cannot clarify these causes of weight loss, which might account for the lack of precise association between BMI at diagnosis and the risk of mortality, which is a limitation of our study.

To date, inconsistent data exist regarding whether the association of BMI with CRC-specific survival rates might differ by patient gender [25,26,47]. Polednak et al. performed a meta-analysis involving 153,760 U.S. patients [47]. They showed that the risk ratio of CRC occurrence was 1.4 for obese men and 1.1 for obese women. This also showed that this affiliation was weaker for women in comparison with men. Slattery et al. [48] showed that patient gender was related to the colon tumorigenesis molecular pathway in that CRC with defective DNA mismatch repair (MMR) was more prevalent in elderly women but not in men. They also observed that an excess of CRCs with defective MMR in elderly women in their study was related to estrogen withdrawal. Sinicrope et al. [26] also remarked that obese patients had significantly fewer CRCs with defective MMR than normal weight patients did.

Another mechanism regarding this discrepancy may be associated with body fat distribution in that a higher BMI is more commonly associated with a higher ratio of abdominal or central adiposity in men [49]. Abdominal adiposity is closely linked with hyperinsulinemia, insulin resistance, and the IGF axis as potential mediators of increased CRC risk and related mortality [50,51]. The lessened impact of obesity on CRC prognosis observed in women vs. men may be related to estrogen effect modifications. Estrogen levels are correlated with BMI in postmenopausal women because their primary source is androgen conversion in adipose tissue [52]. However, this relationship of obesity with CRC risk is lessened after menopause [53], and hormone replacement therapy (HRT) is consistently related to decreased CRC-related mortality rates [54]. Obesity correlates with increased circulating estrogen levels, and estrogen might protect against CRC development in women with defective MMR [48]. Meanwhile, a profound interaction was noticed between underweight status and patient gender, where underweight men had a 39% increase in all-cause mortality compared to normal weight men or women [55]. In our results, a similar phenomenon is demonstrated in Table 2.

In a population-based, case-control study, Russo et al. [56] pointed out that both obesity and physical activity were related to a lower prevalence of CRCs with defective MMR in women but not in men. However, the association between physical activity and cancer is debatable. Although 13–14% of CRC cases may be related to physical inactivity in the U.S. [57], other studies have showed that body weight loss was not sufficient to reduce cancer risk [58]. Physical inactivity deteriorates adipose tissue accumulation, and the subsequent activation of inflammatory cytokines could promote CRC development [59]. Thus, exercise and reducing body fat could alleviate the influence of these factors. A healthier lifestyle, including effective exercise and maintaining a healthy body weight, should be actively promoted to improve a patient’s prognosis [60]. However, the issue of physical activity could not be analyzed in our nationwide population database.

Comorbidity is defined as other underlying diseases in addition to an index disease (e.g., CRC) of primary interest [61]. Comorbid conditions in oncologic patients influence the timing of cancer detection, treatment, prognosis, and outcomes [62]. As others have assumed, comorbidity may be partly accountable for the decreased survival rate noticed in African Americans [63]. Since comorbid conditions apply their effects at multiple levels of oncologic patient care, the failure to explain comorbidity in the studies could confound bias [64]. In Table 1, the underweight patients were noted to have a higher ratio of comorbidities, such as myocardial infarction, cardiovascular disease, dementia, rheumatic disease, peptic ulcer disease, hemiplegia, or paraplegia. These all may contribute to their poorer oncologic outcomes and their increased mortality risk.

There were strengths and limitations of our study. The strengths were the sizeable nationwide sample size, the precise record of BMI measurements, the data on treatment and comorbidities, and the accurate collection of data on survival and recurrence over a prolonged follow-up period of 7 years. The limitations included the retrospective design and the sole measurement of obesity by BMI. BMI alone may not contribute sufficiently accurate information to distinguish body fat and lean mass and fat distribution, and these particulars are significantly different based on age, gender, geographic region, and ethnicity. We were also unable to analyze other parameters such as physical activity, diet, menopausal condition, or HRT use, which might be independently associated with the oncologic outcomes and the mortality risk from other causes. Another major limitation was the probability of reverse causation, which might preclude us from determining a pure group of healthy normal weight people. We might have underestimated the true impact of obesity on oncologic outcomes, since the advancing cancer condition typically leads to weight loss instead of weight gain. Besides this, BMI pre-diagnosis and at diagnosis are correlated with each other, and it is therefore hard to predict the precise time when obesity is acting. However, since the pre-diagnosis BMI is an essential determinant of the BMI at diagnosis, it is strongly recommended that long-term body weight within the normal range is maintained, rather than counting on weight loss after a cancer diagnosis.

## 5. Conclusions

In conclusion, an underweight BMI is correlated with an increased mortality rate in CRC survivors, particularly in men. In underweight patients, a higher recurrence ratio and reduced rates of DFS, CRC-specific survival, and OS suggest increased tumor aggressiveness. Together, these data lead us to propose that active management to improve an underweight BMI after a CRC diagnosis can improve patient outcomes. Future meta-analysis studies should perform more stratified analyses to investigate various populations and examine the impacts on other possible adjustment factors.

## Figures and Tables

**Table 1:** Comparison of the demographic characteristics and underlying comorbidities among different BMI groups with CRC.

Characteristic	Total (*n* = 41,015)	<18.50 (*n* = 2867)	18.50–24.99 (*n* = 23,775)	25.00–29.99 (*n* = 11,741)	30.00–34.99 (*n* = 2246)	≥35.00 (*n* = 386)	*p*-Value
**Gender, *n* (%)**							
Male	23,230 (56.64)	1412 (49.25)	13,261 (55.78)	7200 (61.32)	1191 (53.03)	166 (43.01)	<0.0001
Female	17,785 (43.36)	1455 (50.75)	10,514 (44.22)	4541 (38.68)	1055 (46.97)	220 (56.99)	
**Age Group, *n* (%)**							
<40	1349 (3.29)	154 (5.37)	758 (3.19)	309 (2.63)	98 (4.36)	30 (7.77)	<0.0001
40–49	3468 (8.46)	209 (7.29)	2081 (8.75)	893 (7.61)	236 (10.51)	49 (12.69)	
50–59	8737 (21.30)	472 (16.46)	4909 (20.65)	2727 (23.23)	522 (23.24)	107 (27.72)	
60–69	10,474 (25.54)	498 (17.37)	5816 (24.46)	3395 (28.92)	658 (29.30)	107 (27.72)	
≥70	16987 (41.42)	1534 (53.51)	10,211 (42.95)	4417 (37.62)	732 (32.59)	93 (24.09)	
**Clinic Stage, *n* (%)**							
0	762 (1.82)	40 (1.40)	413 (1.74)	239 (1.89)	54 (2.40)	16 (4.15)	<0.0001
1	9220 (22.00)	448 (15.63)	4929 (20.73)	3069 (24.28)	669 (29.79)	105 (27.20)	
2	8667 (20.68)	655 (22.85)	4561 (19.18)	2957 (23.39)	406 (18.08)	88 (22.80)	
3	15,203 (36.27)	1004 (35.02)	8829 (37.14)	4468 (35.35)	786 (35.00)	116 (30.05)	
4	8063 (19.24)	720 (25.11)	5043 (21.21)	1908 (15.09)	331 (14.74)	61 (15.80)	
**Site, *n* (%)**							
Malignant neoplasm of colon	26,153 (63.76)	1847 (64.42)	15,293 (64.32)	7301 (62.18)	1448 (64.47)	264 (68.39)	0.0073
Rectosigmoid junction	3441 (8.39)	234 (8.16)	1975 (8.31)	1015 (8.64)	188 (8.37)	29 (7.51)	
Rectum	11,421 (27.85)	786 (27.42)	6507 (27.37)	3425 (29.17)	610 (27.16)	93 (24.09)	
**Treatment, *n* (%)**							
Operation	37,240 (90.80)	2401 (83.75)	21,486 (90.37)	10,886 (92.72)	2111 (93.99)	356 (92.23)	<0.0001
Radiotherapy	4782 (11.66)	323 (11.27)	2827 (11.89)	1366 (11.63)	221 (9.84)	45 (11.66)	0.0638
Chemotherapy	24,760 (60.37)	1572 (54.83)	14,618 (61.48)	7063 (60.16)	1292 (57.52)	215 (55.70)	<0.0001
**Smoking, *n* (%)**							
Never	29,872 (72.83)	2070 (72.20)	17,428 (73.30)	8396 (71.51)	1675 (74.58)	303 (78.50)	<0.0001
Quit	4415 (10.76)	280 (9.77)	2447 (10.29)	1410 (12.01)	249 (11.09)	29 (7.51)	
Current	6728 (16.40)	517 (18.03)	3900 (16.40)	1935 (16.48)	322 (14.34)	54 (13.99)	
Death, *n* (%)	15,021 (36.62)	1617 (56.40)	9097 (38.26)	3550 (30.24)	655 (29.16)	102 (26.42)	<0.0001
Time to mortality, Median (Q1-Q3)	1.49 (0.64–2.62)	0.97 (0.37–2.01)	1.48 (0.64–2.58)	1.71 (0.79–2.88)	1.82 (0.88–2.96)	1.69 (0.79–2.92)	<0.0001
Death in colon cancer, *n* (%)	11,347 (27.67)	1186 (41.37)	6911 (29.07)	2696 (22.96)	483 (21.50)	71 (18.39)	<0.0001
Time to specific-mortality, Median (Q1-Q3)	1.41 (0.61–2.43)	0.93 (0.33–1.88)	1.38 (0.60–2.38)	1.62 (0.77–2.65)	1.77 (0.88–2.86)	1.53 (0.76–2.83)	<0.0001
Recurrence, *n* (%)	2068 (5.04)	153 (5.34)	1218 (5.12)	576 (4.91)	106 (4.72)	15 (3.89)	0.0016
Time to recurrence, Median (Q1-Q3)	0.64 (0.44–0.84)	0.56 (0.41–0.78)	0.64 (0.44–0.83)	0.65 (0.45–0.85)	0.63 (0.46–0.85)	0.69 (0.41–1.00)	0.0402
**Comorbidity, *n* (%)**							
Myocardial infarction	482 (1.18)	41 (1.43)	238 (1.00)	164 (1.40)	35 (1.56)	4 (1.01)	0.0035
Congestive heart failure	1977 (4.82)	152 (5.30)	1044 (4.39)	588 (5.01)	167 (7.44)	26 (6.74)	<0.0001
Peripheral vascular disease	752 (1.83)	51 (1.78)	433 (1.82)	219 (1.87)	41 (1.83)	8 (2.07)	0.9924
Cardiovascular disease	3396 (8.28)	289 (10.08)	1975 (8.31)	930 (7.92)	184 (8.19)	18 (4.66)	0.0003
Dementia	921 (2.25)	161 (5.62)	569 (2.39)	170 (1.45)	20 (0.89)	1 (0.26)	<0.0001
COPD	3339 (8.14)	307 (10.71)	1816 (7.64)	951 (8.10)	213 (9.48)	52 (13.47)	<0.0001
Rheumatic disease	432 (1.05)	33 (1.15)	261 (1.10)	121 (1.03)	13 (0.58)	4 (1.04)	0.2289
peptic ulcer disease	5966 (14.55)	514 (17.93)	3462 (14.56)	1625 (13.84)	297 (13.22)	68 (17.62)	<0.0001
Mild liver disease	3151 (7.68)	196 (6.84)	1702 (7.16)	1006 (8.57)	212 (9.44)	35 (9.07)	<0.0001
Diabetes without chronic complication	8805 (21.47)	362 (12.63)	4630 (19.47)	2992 (25.48)	682 (30.37)	139 (36.01)	<0.0001
Diabetes with chronic complication	2180 (5.32)	77 (2.69)	1123 (4.72)	762 (6.49)	184 (8.19)	34 (8.81)	<0.0001
Hemiplegia or paraplegia	287 (0.70)	27 (0.94)	175 (0.74)	70 (0.60)	12 (0.53)	3 (0.78)	0.2313
Renal disease	2188 (5.33)	144 (5.02)	1244 (5.23)	635 (5.41)	146 (6.50)	19 (4.92)	0.1187
Moderate or severe liver disease	157 (0.38)	8 (0.28)	89 (0.37)	48 (0.41)	11 (0.49)	1 (0.26)	0.7557
**Grade, *n* (%)**							
1. Well differentiated	3266 (7.96)	191 (6.66)	1859 (7.96)	974 (8.30)	202 (8.99)	40 (10.36)	<0.0001
2. Moderately differentiated	33,134 (80.79)	2312 (80.64)	19,139 (80.38)	9571 (81.52)	1811 (80.63)	301 (77.98)	
3. Poorly differentiated	3734 (9.10)	285 (9.94)	2268 (9.53)	969 (8.25)	176 (7.84)	36 (9.33)	
4. Undifferentiated	393 (0.96)	33 (1.15)	234 (0.98)	103 (0.88)	20 (0.89)	3 (0.78)	
5. Others	488 (1.19)	46 (1.60)	275 (1.15)	124 (1.06)	37 (1.65)	6 (1.55)	

*p.s.* The time of median follow up was 3.27 (2.08–4.82) years.

**Table 2:** Hazard Ratios of BMI at Diagnosis and Mortality Rates.

Characteristic	Patient (*n* = 41015)	Event (*n* = 15021)	Age-, Gender-Adjusted HR	*p*-Value	Age-, Gender-,Stage- Adjusted HR	*p*-Value	Multivariable-Adjusted HR ^†^	*p*-Value
**Overall Survival**								
**BMI WHO Categories, *n* (%)**								
<18.50	2867	1617 (56.40)	1.76 (1.67–1.85)	<0.0001	1.68 (1.59–1.77)	<0.0001	1.61 (1.53–1.70)	<0.0001
18.50–24.99	23,775	9097 (38.26)	Ref.		Ref.		Ref.	
25.00–29.99	11,741	3550 (30.24)	0.75 (0.72–0.78)	<0.0001	0.82 (0.79–0.86)	<0.0001	0.82 (0.79–0.85)	<0.0001
30.00–34.99	2246	655 (29.16)	0.76 (0.71–0.83)	<0.0001	0.87 (0.80–0.94)	<0.0001	0.83 (0.77–0.90)	<0.0001
≥35.00	386	102 (26.42)	0.73 (0.60–0.89)	0.0018	0.83 (0.68–1.01)	<0.0001	0.74 (0.61–0.90)	0.0027
**CRC-Specific Survival**								
**BMI WHO Categories, *n* (%)**								
<18.50	2867	1186 (41.37)	1.69 (1.59–1.79)	<0.0001	1.59 (1.50–1.69)	<0.0001	1.52 (1.43–1.62)	<0.0001
18.50–24.99	23,775	6911 (29.07)	Ref.		Ref.		Ref.	
25.00–29.99	11,741	2696 (22.96)	0.75 (0.72–0.79)	<0.0001	0.85 (0.81–0.88)	<0.0001	0.85 (0.81–0.89)	<0.0001
30.00–34.99	2246	483 (21.50)	0.73 (0.66–0.80)	<0.0001	0.86 (0.79–0.95)	0.0015	0.85 (0.78–0.94)	0.0009
≥35.00	386	71 (18.39)	0.64 (0.50–0.80)	0.0001	0.74 (0.59–0.94)	0.0119	0.69 (0.54–0.87)	0.0017
**Disease Free Survival ^#^**								
**BMI WHO categories, *n* (%)**								
<18.50	1853	737 (39.77)	1.73 (1.60–1.87)	<0.0001	1.71 (1.58–1.85)	<0.0001	1.75 (1.62–1.89)	<0.0001
18.50–24.99	17,660	4454 (25.22)	Ref.		Ref.		Ref.	
25.00–29.99	9417	1932 (20.52)	0.81 (0.77–0.85)	<0.0001	0.83 (0.78–0.87)	<0.0001	0.81 (0.77–0.86)	<0.0001
30.00–34.99	1825	381 (20.88)	0.89 (0.80–0.99)	0.0329	0.94 (0.84–1.04)	0.2212	0.87 (0.79–0.97)	0.0122
≥35.00	308	53 (17.21)	0.79 (0.60–1.03)	0.0797	0.83 (0.64–1.09)	0.1852	0.75 (0.57–0.98)	0.0351

^†^ Multivariable-adjusted HRs were adjusted for age, gender, stage, grade, cancer site, treatment types, smoking status, and comorbidities. ^#^ Excluding 9952 patients whose cancer recurrence were unknown.

**Table 3:** Stratified analyses of overall survival rates.

Characteristic	BMI WHO Categories				
	<18.50	18.50–24.99	25.00–29.99	30.00–34.99	≥35.00
**Age Group**					
<40, No	154	758	309	98	30
Event, No	51	251	85	34	9
HR (95% CI)	0.96 (0.70–1.31)	Ref.	0.96 (0.74–1.24)	1.14 (0.79–1.65)	0.69 (0.35–1.37)
40–49, No	209	2081	893	236	49
Event, No	86	595	238	48	9
HR (95% CI)	1.23 (0.98–1.54)	Ref.	1.03 (0.88–1.20)	0.81 (0.60–1.09)	0.77 (0.40–1.50)
50–59, No	472	4909	2727	522	107
Event, No	194	1407	638	127	25
HR (95% CI)	1.49 (1.28–1.73) **	Ref.	0.84 (0.76–0.92) *	1.01 (0.84–1.22)	0.76 (0.51–1.14)
60–69, No	498	5816	3395	658	107
Event, No	237	1728	821	149	20
HR (95% CI)	1.85 (1.61–2.13) **	Ref.	0.90 (0.83–0.98) *	0.88 (0.75–1.05)	0.85 (0.55–1.32)
≥70, No	1534	10,211	4417	732	93
Event, No	1049	5116	1768	297	39
HR (95% CI)	1.62 (1.51–1.73) **	Ref.	0.77 (0.73–0.81) **	0.77 (0.69–0.87) **	0.75 (0.54–1.02)
**Gender**					
Male, No	1412	13,261	7200	1191	166
Event, No	888	5436	2210	361	46
HR (95% CI)	1.72 (1.60–1.85) **	Ref.	0.79 (0.75–0.83) **	0.84 (0.76–0.94) *	0.79 (0.59–1.06)
Female, No	1455	10,514	4541	1055	220
Event, No	729	3661	1340	294	56
HR (95% CI)	1.48 (1.36–1.60) **	Ref.	0.85 (0.80–0.91) **	0.81 (0.72–0.92) *	0.69 (0.53–0.90) *
**Clinic Stage**					
Stage 0, No	40	413	239	54	16
Event, No	18	57	15	3	1
HR (95% CI)	3.72 (1.95–7.11) **	Ref.	0.45 (0.25–0.82) *	0.32 (0.09–1.09)	0.43 (0.05–3.53)
Stage 1, No	448	4929	3069	669	105
Event, No	171	937	412	102	13
HR (95% CI)	2.23 (1.88–2.64) **	Ref.	0.69 (0.61–0.78) **	0.85 (0.69–1.04)	0.72 (0.41–1.24)
Stage 2, No	655	4561	2957	406	88
Event, No	321	1340	483	87	18
HR (95% CI)	1.94 (1.71–2.19) **	Ref.	0.74 (0.67–0.82) **	0.72 (0.58–0.89) *	0.75 (0.47–1.21)
Stage 3, No	1004	8829	4468	786	116
Event, No	487	2699	1144	206	29
HR (95% CI)	1.85 (1.68–2.04) **	Ref.	0.82 (0.77–0.88) **	0.88 (0.76–1.01)	0.90 (0.62–1.30)
Stage 4, No	720	5043	1908	331	61
Event, No	620	4064	1496	257	41
HR (95% CI)	1.23 (1.13–1.34) **	Ref.	0.90 (0.85–0.96) *	0.90 (0.79–1.02)	0.68 (0.50–0.93) *
**Site**					
Malignant neoplasm of colon, No	1847	15,293	7301	1448	264
Event, No	1018	5853	2215	428	72
HR (95% CI)	1.56 (1.46–1.67) **	Ref.	0.82 (0.78–0.86) **	0.84 (0.76–0.93) *	0.84 (0.67–1.06)
Rectosigmoid junction, No	234	1975	1015	188	29
Event, No	137	757	294	53	5
HR (95% CI)	1.66 (1.38–2.01) **	Ref.	0.81 (0.71–0.93) *	0.79 (0.60–1.05)	0.32 (0.13–0.78) *
Rectum, No	786	6507	3425	610	93
Event, No	462	2487	1041	174	25
HR (95% CI)	1.78 (1.61–1.97) **	Ref.	0.82 (0.76–0.88) **	0.83 (0.71–0.97) *	0.70 (0.47–1.03)

* *p* < 0.05, ** *p* < 0.0001.

**Table 4:** Stratified analyses of disease-free survival rates.

Characteristic	WHO Categories				
	<18.50	18.50–24.99	25.00–29.99	30.00–34.99	≥35.00
**Age Group**					
<40, No	104	528	233	73	24
Event, No	18	84	38	16	4
HR (95% CI)	1.06 (0.63–1.79)	Ref.	1.05 (0.71–1.57)	1.41 (0.81–2.44)	0.86 (0.30–2.42)
40–49, No	142	1561	696	193	37
Event, No	33	232	105	28	2
HR (95% CI)	1.30 (0.89–1.90)	Ref.	1.07 (0.84–1.36)	0.95 (0.64–1.42)	0.35 (0.09–1.42)
50–59, No	337	3760	2221	436	86
Event, No	83	629	325	75	14
HR (95% CI)	1.50 (1.19–1.90) *	Ref.	0.88 (0.77–1.00) *	1.05 (0.82–1.34)	1.05 (0.61–1.80)
60–69, No	330	4498	2842	554	89
Event, No	95	796	446	88	11
HR (95% CI)	1.92 (1.55–2.38) **	Ref.	0.88 (0.79–0.99) *	0.92 (0.74–1.15)	0.76 (0.42–1.37)
≥70, No	940	7313	3425	569	72
Event, No	508	2713	1018	174	22
HR (95% CI)	1.79 (1.62–1.97) **	Ref.	0.75 (0.70–0.81) **	0.79 (0.67–0.92) *	0.73 (0.48–1.11)
**Gender**					
Male, No	878	9721	5728	970	139
Event, No	423	2725	1165	209	29
HR (95% CI)	1.88 (1.70–2.09) **	Ref.	0.75 (0.70–0.80) **	0.84 (0.73–0.97) *	0.82 (0.57–1.19)
Female, No	975	7939	3689	855	169
Event, No	314	1729	767	172	24
HR (95% CI)	1.59 (1.41–1.80) **	Ref.	0.92 (0.85–1.00) *	0.93 (0.79–1.08)	0.71 (0.47–1.06)
**Clinic Stage**					
Stage 0, No	34	385	223	49	15
Event, No	12	49	14	2	1
HR (95% CI)	3.24 (1.52–6.90) *	Ref.	0.41 (0.22–0.77) *	0.20 (0.04–0.93) *	0.27 (0.03–2.35)
Stage 1, No	388	4619	2906	641	96
Event, No	128	818	365	100	9
HR (95% CI)	2.06 (1.70–2.49) **	Ref.	0.69 (0.61–0.78) **	0.92 (0.74–1.13)	0.56 (0.29–1.07)
Stage 2, No	540	4065	1880	371	80
Event, No	232	1082	417	76	18
HR (95% CI)	1.85 (1.61–2.14) **	Ref.	0.78 (0.70–0.88) **	0.75 (0.59–0.94) *	0.95 (0.60–1.52)
Stage 3, No	798	7780	4055	703	101
Event, No	319	2130	988	177	20
HR (95% CI)	1.64 (1.46–1.85) **	Ref.	0.88 (0.81–0.95) *	0.94 (0.81–1.10)	0.77 (0.50–1.20)
Stage 4, No	93	811	353	61	16
Event, No	46	375	148	26	5
HR (95% CI)	1.23 (0.90–1.68)	Ref.	0.89 (0.73–1.08)	0.85 (0.57–1.27)	0.68 (0.28–1.69)
**Site**					
Malignant neoplasm of colon, No	1248	11397	5850	1169	212
Event, No	492	2856	1156	233	39
HR (95% CI)	1.69 (1.54–1.87) **	Ref.	0.78 (0.73–0.84) **	0.83 (0.73–0.96) *	0.89 (0.65–1.23)
Rectosigmoid junction, No	151	1480	851	147	26
Event, No	67	372	192	28	3
HR (95% CI)	1.99 (1.53–2.60) **	Ref.	0.91 (0.76–1.09)	0.81 (0.55–1.19)	0.21 (0.06–0.67) *
Rectum, No	454	4783	2716	509	70
Event, No	178	1226	584	120	11
HR (95% CI)	1.83 (1.56–2.15) **	Ref.	0.84 (0.76–0.93) *	1.00 (0.83–1.20)	0.65 (0.36–1.18)

* *p* < 0.05, ** *p* < 0.0001.

**Table 5:** Stratified analyses of CRC-specific survival rates.

Characteristic	WHO Categories				
	<18.50	18.50–24.99	25.00–29.99	30.00–34.99	≥35.00
**Age Group**					
<40, No	154	758	309	98	30
Event, No	49	236	79	31	9
HR (95% CI)	1.00 (0.73–1.38)	Ref.	0.94 (0.72–1.23)	1.11 (0.75–1.64)	0.76 (0.38–1.50)
40–49, No	209	2081	893	236	49
Event, No	78	543	218	41	6
HR (95% CI)	1.24 (0.97–1.57)	Ref.	1.06 (0.90–1.24)	0.82 (0.59–1.13)	0.57 (0.25–1.27)
50–59, No	472	4909	2727	522	107
Event, No	158	1214	547	101	19
HR (95% CI)	1.38 (1.17–1.64) *	Ref.	0.87 (0.78–0.96) *	1.04 (0.85–1.28)	0.68 (0.43–1.08)
60–69, No	498	5816	3395	658	107
Event, No	183	1370	644	117	12
HR (95% CI)	1.76 (1.51–2.06) **	Ref.	0.94 (0.86–1.04)	0.96 (0.79–1.16)	0.73 (0.41–1.29)
≥70, No	1534	10,211	4417	732	93
Event, No	718	3548	1208	193	25
HR (95% CI)	1.54 (1.42–1.67) **	Ref.	0.79 (0.74–0.84) **	0.76 (0.66–0.88) *	0.74 (0.50–1.10)
**Gender**					
Male, No	1412	13261	7200	1191	166
Event, No	616	3973	1678	252	28
HR (95% CI)	1.62 (1.49–1.77) **	Ref.	0.84 (0.80–0.89) **	0.85 (0.75–0.97) *	0.69 (0.47–1.00) *
Female, No	1455	10,514	4541	1055	220
Event, No	570	2938	1018	231	43
HR (95% CI)	1.43 (1.30–1.56) **	Ref.	0.85 (0.79–0.92) **	0.84 (0.74–0.96) *	0.67 (0.49–0.90) *
**Clinic Stage**					
Stage 0, No	40	413	239	54	16
Event, No	4	11	2	1	1
HR (95% CI)	9.29 (1.42–60.60) *	Ref.	0.29 (0.06–1.51)	0.21 (0.02–3.00)	4.24 (0.34–52.80)
Stage 1, No	448	4929	3069	669	105
Event, No	77	421	187	43	4
HR (95% CI)	2.15 (1.67–2.76) **	Ref.	0.71 (0.60–0.85) *	0.84 (0.61–1.15)	0.49 (0.18–1.30)
Stage 2, No	655	4561	2957	406	88
Event, No	198	822	303	51	6
HR (95% CI)	1.95 (1.67–2.28) **	Ref.	0.77 (0.68–0.88) *	0.71 (0.54–0.95) *	0.40 (0.18–0.90) *
Stage 3, No	1004	8829	4468	786	116
Event, No	347	1970	833	155	20
HR (95% CI)	1.83 (1.63–2.05) **	Ref.	0.83 (0.76–0.90) **	0.91 (0.77–1.07)	0.84 (0.54–1.31)
Stage 4, No	720	5043	1908	331	61
Event, No	560	3687	1371	233	40
HR (95% CI)	1.23 (1.13–1.35) **	Ref.	0.92 (0.86–0.98) *	0.90 (0.79–1.03)	0.73 (0.53–1.00) *
**Site**					
Malignant neoplasm of colon, No	1847	15,293	7301	1448	264
Event, No	737	4449	1670	308	52
HR (95% CI)	1.45 (1.34–1.57) **	Ref.	0.85 (0.80–0.90) **	0.85 (0.75–0.95) *	0.79 (0.60–1.04)
Rectosigmoid junction, No	234	1975	1015	188	29
Event, No	103	587	219	41	2
HR (95% CI)	1.57 (1.26–1.96) **	Ref.	0.79 (0.68–0.93) *	0.82 (0.59–1.12)	0.19 (0.05–0.77) *
Rectum, No	786	6507	3425	610	93
Event, No	346	1875	807	134	17
HR (95% CI)	1.73 (1.54–1.95) **	Ref.	0.86 (0.79–0.93) *	0.89 (0.75–1.06)	0.65 (0.40–1.06)

* *p* < 0.05, ** *p* < 0.0001.

## Data Availability

The data sources are the Taiwan Nation Health Insurance Database and Taiwan Cancer Registry. The data are available with the permission from Taiwan Health and Welfare Data Science Centre (https://dep.mohw.gov.tw/DOS/np-2497-113.html, accessed on 5 May 2021). Restrictions apply to the availability of these data, which were used under license for this study.
